# Excessive Daytime Sleepiness Should Be Systematically Assessed in Individuals With Insomnia: A Population‐Based Study Employing a Virtual Agent‐Based Digital Tool

**DOI:** 10.1111/jsr.70178

**Published:** 2025-08-28

**Authors:** Julien Coelho, Florian Pécune, Alex Chanteclair, Christophe Gauld, Etienne de Sevin, Emmanuel d'Incau, Patricia Sagaspe, Tafsir Ka, Hervé Alia, Charles M. Morin, Jean‐Arthur Micoulaud‐Franchi, Pierre Philip

**Affiliations:** ^1^ Univ. Bordeaux, SANPSY, UMR 6033 Bordeaux France; ^2^ CHU Bordeaux, Service Universitaire de Médecine du Sommeil Bordeaux France; ^3^ CH Périgueux Périgueux France; ^4^ École de Psychologie Université Laval Québec Canada; ^5^ Centre de recherche CERVO/BRAIN Research Center Université Laval Québec Canada

**Keywords:** cohort, conversational agents, excessive daytime sleepiness, insomnia

## Abstract

Insomnia and excessive daytime sleepiness (EDS) often co‐occur, despite involving distinct physiological mechanisms. The KANOPÉE application, a fully autonomous virtual agent that collects sleep‐related data and delivers personalised behavioural recommendations over a 17‐day period, offers a unique opportunity to better understand this unexpected phenotype. Our primary aim was to characterise these ‘sleepy insomniacs’, and our secondary aim was to evaluate their response to this digital sleep intervention. Among 21,590 participants, individuals with an Epworth Sleepiness Scale score ≥ 11 and an Insomnia Severity Index score ≥ 15 were classified as ‘sleepy insomniacs’. Comorbidities (i.e., obstructive sleep apnea syndrome, restless legs syndrome, depression, and sleep medication use) were first described and then excluded for further analyses. At baseline, 4843 (47.9%) of the 10,114 participants with insomnia also reported EDS and were categorised as ‘sleepy insomniacs’. Half of this subgroup reported at least one comorbidity, with depression being the most common. After excluding participants with comorbidities, 3239 individuals (44.3%) remained in the ‘sleepy insomniacs’ category. These individuals were more likely to experience middle or late insomnia symptoms compared to those with insomnia without EDS but responded similarly to the digital sleep intervention. In conclusion, EDS is highly prevalent among individuals with insomnia symptoms. While comorbidities, particularly depression, explained the co‐occurrence in approximately half of the sample, a substantial proportion of participants without comorbidities also exhibited this unexpected phenotype. The association with specific insomnia subtypes highlights the need for further investigation. Notably, a 17‐day digital sleep intervention proved effective in treating ‘sleepy insomniacs’.

## Graphical abstract

In a cohort of 21,590 participants undergoing a 17‐day digital sleep intervention, the ‘sleepy insomniac’ phenotype was highly prevalent and was associated with comorbidities in about half of the cases. Compared to individuals with insomnia without sleepiness, these participants were more likely to experience middle or late insomnia symptoms but showed a similar response to the digital sleep intervention.

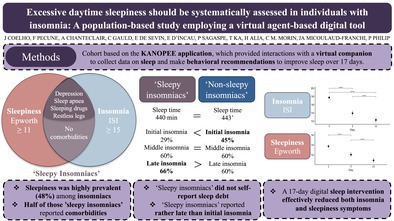

## 1 Introduction

Insomnia and excessive daytime sleepiness (EDS) are the two most common sleep complaints and are highly prevalent in the general population (Morin and Jarrin 2022). Both conditions are associated with various physical and mental health outcomes (Bock et al. 2022; Chalet et al. 2023), including an increased risk of driving accidents (Bioulac et al. 2017). These complaints may arise from sleep disorders (American Academy of Sleep Medicine 2014) as well as inappropriate behaviours (Buysse 2014), such as staying in bed while awake in the case of insomnia (Birling et al. 2020) or sleep deprivation in the case of EDS (Cellini et al. 2023). Findings regarding their association remain inconsistent (Kolla et al. 2020; Shekleton et al. 2010; Zhang et al. 2022). Nevertheless, their co‐occurrence in certain individuals is undeniable, despite the fact that they involve distinct and sometimes opposing physiological mechanisms (American Academy of Sleep Medicine 2014; Fasiello et al. 2024; Hein et al. 2017; Riemann et al. 2010).

The co‐occurrence of insomnia and EDS may be attributed to comorbidities, such as sleep disorders (e.g., obstructive sleep apnea syndrome [OSAS] or restless legs syndrome [RLS]) (American Academy of Sleep Medicine 2014) and psychiatric disorders (e.g., major depressive disorder) (American Psychiatric Association 2017). The COMorbid Insomnia and Sleep Apnea (COMISA) study, a preferred model for investigating the relationship between insomnia and EDS (Sweetman et al. 2023), suggested that factors such as night‐to‐day variability in each symptom, the side effects of sleeping pills and/or stimulants, and environmental conditioning (e.g., arousal in bed or sleepiness while watching TV) might contribute to their unexpected co‐occurrence in other conditions (Sweetman et al. 2021). Above all, EDS can be particularly difficult to distinguish from fatigue, a related but distinct construct characterised by a lack of physical or mental energy rather than a tendency to fall asleep, especially in patients with insomnia symptoms (Shen et al. 2006). Despite these insights, the complex relationship between insomnia and EDS has yielded limited and conflicting results to date.

Due to the limited availability of educational programs in France and abroad for treating sleep disturbances resulting from inappropriate behaviours (Shelgikar 2024), we developed a research program utilising virtual agents to autonomously address and treat the poor sleep behaviours occurring on a large scale within the general population (Dupuy et al. 2021; Philip et al. 2020). To date, 60,000 individuals have downloaded the free app (KANOPEE) from the Google or Apple Store to access sleep hygiene and stimulus control recommendations. Our findings demonstrate that these autonomous behavioural interventions significantly improved sleep schedules and reduced the overall severity of insomnia symptoms (Philip et al. 2022; Philip et al. 2020). Participants were also monitored for symptoms of depression, a prevalent and detrimental condition (Palagini et al. 2022) that is strongly associated with sleep behaviours and complaints (Alvaro et al. 2013; Riemann 2018).

This digital sleep cohort offers a unique opportunity to gain a deeper understanding of the co‐occurrence of insomnia and EDS. Thus, our aim was to take an exploratory approach to describe the sociodemographic characteristics, comorbidities, and sleep behaviours of participants exhibiting this unexpected phenotype, as well as to evaluate the efficacy of a digital sleep intervention for these individuals.

## 2 Methods

### 2.1 Study Design, Setting, and Participants

This ancillary study was conducted within the population cohort engaged in the KANOPEE application, which offers interactions with a virtual companion to collect sleep data and provide personalised behavioural recommendations over a 17‐day period. The application was promoted through various platforms, including social media (Instagram, TikTok, Facebook, and Twitter), national and regional newspapers, and television, as well as university and hospital mailing lists, to increase download rates and reach a diverse population. The application was not advertised, and participants were not compensated. Eligibility criteria included downloading KANOPEE, completing the baseline interview, and being of legal age (≥ 18 years). Recruitment occurred via the Google Play Store or the Apple Store, where participants could download the app. Informed consent was provided directly through the app and was required from all participants before any data collection could occur. Approval from the scientific committees of the University of Bordeaux was granted, and the study was in compliance with the General Data Protection Regulation, with authorisation from the French authorities (CNIL). Study method and results are reported following the Strengthening the Reporting of Observational Studies in Epidemiology (STROBE) Statement for observational studies (von Elm et al. 2014).

**FIGURE 1:**
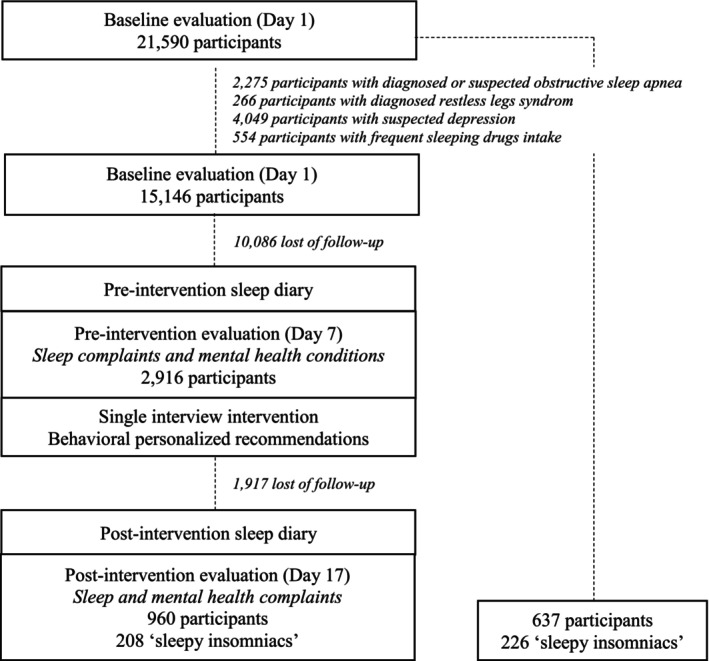
Flowchart of participant inclusion through the KANOPEE application.

This was a single‐arm intervention using different steps (Figure 1) and previously described (Philip et al. 2022, 2020). First, on day 1, participants interacted with a virtual companion who provided screening for sociodemographic characteristics, comorbidities, and baseline sleep complaints. The assessments were completed by 21,590 participants between 27 July 2022 and 6 October 2024. Second, participants were asked to complete a pre‐intervention sleep diary between day 1 and day 7 to evaluate their sleep behaviours. A minimum of 7 days was completed in accordance with recommendations on subjective sleep measurements of sleep duration and regularity (Aili et al. 2017; Fischer et al. 2021). Third, on day 7, participants met the virtual companion again who conducted a pre‐intervention interview (i.e., pre‐intervention assessment of sleep complaints). The virtual companion provides personalised behavioural recommendations according to sleep diary data and sleep complaint answers. The intervention was delivered in a single 20‐min interview and focused on reinforcing rise time regularity, adjusting the estimated sleep duration with time spent in bed, and developing appropriate behaviours to reinforce circadian rhythms. For example, participants reporting sleep maintenance insomnia symptoms during interviews or prolonged awakenings during the night in the sleep diary were encouraged to apply the stimulus control guideline. The recommendations were simple sentences (e.g., ‘If I'm awake for more than 15 min at night, I get out of bed and only return to bed when I'm sleepy’). The precise set of instructions along with the detailed algorithm of personalisation are available in Table S1. After the intervention was delivered, a new tab ‘personalised recommendations’ appeared at the bottom of the screen so that participants could access them at any time. Fourth, participants were encouraged to follow these recommendations for 10 days while completing the sleep diary, and they were informed that they would have a third, final, interview 10 days later. Fifth, on day 17, participants were asked to interact with the virtual companion for a post‐intervention interview and to complete the third and last sleep complaints assessment (*n* = 1597).

### 2.2 Measures

#### 2.2.1 Sociodemographic Characteristics

During the baseline evaluation (Day 1), participants were asked to provide their sociodemographic characteristics, including age (continuous), sex (male or female), educational level (middle school, high school, bachelor's degree, or master's degree), and occupational category. Occupational categories included higher intellectual professions (e.g., engineers and doctors), intermediate professions (e.g., technicians and teachers), employee and worker roles (e.g., administrative agents and maintenance workers), and no professional activity.

#### 2.2.2 Comorbidities

During the baseline evaluation (Day 1), participants were also queried about a previous diagnosis of OSAS or RLS. Suspected OSAS was assessed using the NoSAS score, which evaluates the likelihood of OSAS based on age, sex, overweight status, snoring, and neck circumference (Marti‐Soler et al. 2016). A score of eight or higher was considered suspicious of OSAS. Comorbid depression was measured using the Patient Health Questionnaire (PHQ‐9), a reliable and valid nine‐item tool rated on a 3‐point Likert scale assessing symptoms over the past 2 weeks (Kroenke et al. 2001). The Cronbach's α value in the original study was 0.89, and it was satisfactory in our sample (0.82). A score of ≥ 10 indicated moderate to severe depression. Participants were also asked daily about their use of sleep medications, with consumption considered frequent if it occurred on at least half of the days.

#### 2.2.3 Sleep Complaints

Insomnia and EDS were assessed at baseline (Day 1), pre‐intervention (Day 7), and post‐intervention (Day 17). Insomnia symptoms were evaluated using the Insomnia Severity Index (ISI), a seven‐item scale with each item rated on a 5‐point Likert scale ranging from 0 to 4 (Bastien et al. 2001). The Cronbach's *α* was satisfactory both in the original validation study (0.76) and in our sample (0.80). Participants were then categorised into the following groups: severe insomnia (ISI ≥ 22), moderate insomnia (15 ≤ ISI < 22), subthreshold insomnia (8 ≤ ISI < 15), and no insomnia (ISI < 8). Additionally, the first three items of the ISI, assessing initial insomnia (item 1), middle insomnia (item 2), and late insomnia (item 3), were dichotomised (0–2 vs. 3–4) and analysed separately in secondary analyses. These items were also used to classify insomnia according to the most prominent symptom (initial, middle, or late). In cases of equal severity across symptoms, insomnia was categorised as ‘combined’.

EDS was assessed using the Epworth Sleepiness Scale (ESS), an eight‐item scale where each item is rated on a 4‐point Likert scale ranging from 0 to 3 (Johns 1991). The Cronbach's *α* was good in a transcultural validation study (0.88) (Kaminska et al. 2010), and in our sample (0.80). Participants were categorised into the following groups: severe EDS (ESS ≥ 16), moderate EDS (11 ≤ ESS < 16), and no EDS (ESS < 11). Additionally, items 6 and 8 of the ESS, which are believed to assess active conditions, were summed and analysed separately from the other items, which are thought to assess passive conditions (Pilcher et al. 2018).

Based on their sleep complaints, four participant profiles were defined: severe ‘sleepy insomniacs’ (ISI ≥ 22 and ESS ≥ 16), moderate ‘sleepy insomniacs’ (ISI ≥ 15 and ESS ≥ 11), insomnia without EDS (ISI ≥ 15 and ESS < 11), and healthy participants (ISI < 15 and ESS < 11).

#### 2.2.4 Sleep Behaviours

Participants were asked to complete sleep diaries twice: once between baseline (Day 1) and the pre‐intervention evaluation (Day 7), and again between Day 7 and the post‐intervention evaluation (Day 17). Data from both sleep diaries were used to calculate sleep behaviour parameters, including total sleep time (TST), defined as the average time between falling asleep and the last awakening minus any time reported as awake; the mid‐sleep point (MSP), defined as the average midpoint between first falling asleep and the last awakening; wake after sleep onset (WASO), defined as the time spent awake between falling asleep and the last awakening; and nap duration, defined as the cumulative sleep time during naps. For each metric, intraindividual *means* (IIMs) were calculated by summing daily values and dividing by the total number of days for each participant, and intraindividual standard deviations (ISD) were calculated across daily values using the R package *Varian*. The IIM of TST was used as a proxy for sleep quantity, whereas the IIM of MSP served as a proxy for sleep timing. Finally, the sleep regularity index (SRI), defined as the average probability of an individual being in the same state (sleep or wake) at any two time points 24 h apart, was calculated. The SRI score ranged from 0 (completely random) to 100 (perfectly regular). The SRI and the ISDs of TST and MSP served as proxies for sleep regularity.

### 2.3 Statistical Analysis

The total sample (*n =* 21,590) was utilised for the first part of the analysis. *Frequencies* (%) were calculated and compared using the *Chi‐square test* for categorical variables, whereas *means* and *standard deviations* were computed for continuous variables and compared using the *two‐sample Student's t‐test*. First, differences in sociodemographic characteristics and comorbidities across the four sleep complaint profiles at baseline were analysed. Participants reporting a diagnosis or suspicion of OSAS (NoSAS ≥ 8, *n* = 2275), RLS (*n* = 266), depression (PHQ‐9 *≥* 10, *n* = 4049), or frequent use of sleep medications (*n* = 554) were excluded from further analyses. The sample consisting of participants without comorbidities (*n* = 15,146) was utilised for the second part of the analysis. Differences in sociodemographic characteristics (age and sex), sleep complaints (global and subtypes of insomnia symptoms and EDS) and sleep behaviours (TST, MSP, WASO, Naps, SRI) across the four sleep complaint profiles at baseline were reanalysed within this sample, with a specific comparison made between participants with insomnia symptoms without EDS and ‘sleepy insomniacs’. The sample of ‘sleepy insomniacs’ with complete follow‐up data (*n* = 434) was utilised for the third part of the analysis. Changes in sleep complaints during follow‐up were represented as *means* and *bootstrap confidence intervals* (CIs) at each time point (Days 1, 7, and 17) for ‘sleepy insomniacs’ with comorbidities (*n* = 226) or without comorbidities (*n* = 208). *Cohen's d* effect sizes were also calculated between baseline and post‐intervention evaluations for both sleep and mental health complaints. A *p* value < 0.05 was considered significant for all tests. Data analyses were performed using R v.4.1.2 (R Foundation for Statistical Computing, Vienna, Austria).

## 3 Results

### 3.1 Total Sample (*n* = 21,590)

In total, 21,590 participants met the inclusion criteria (mean age: 49.5 ± 14.7 years; 78.1% female; 37.7% overweight). At baseline, 7527 participants (34.9%) reported neither insomnia symptoms nor EDS, 5271 participants (24.4%) reported insomnia symptoms without EDS, 4550 participants (21.1%) were classified as ‘sleepy insomniacs’ (ISI ≥ 15 and ESS ≥ 11), and 293 participants (1.4%) were categorised as severe ‘sleepy insomniacs’ (ISI ≥ 21 and ESS ≥ 16) (Table 1). In other words, 47.9% of participants with insomnia symptoms (ISI ≥ 15) reported EDS (ESS ≥ 11), compared to 34.4% of participants without insomnia symptoms (*p* < 0.001).

**TABLE 1:** Sociodemographic characteristics and comorbidities of all participants according to sleep complaints.

Variables	All	Healthy participants	Insomnia symptoms without EDS	*p* ^a^	Moderate sleepy insomniacs	*p* ^b^	Severe sleepy insomniacs	*p* ^c^
Baseline, Day 1	21,590	7527	5271		4550		293	
Age (*m* ± sd)	49.5 ± 14.7	50.4 ± 15.3	49.7 ± 14.7	**0.009**	48.3 ± 13.8	**< 0.001**	46 ± 14.3	**< 0.001**
Sex				**< 0.001**		**< 0.001**		**< 0.001**
Female	16,870 (78.1%)	5775 (76.7%)	4337 (82.3%)		3645 (80.1%)		242 (82.6%)	
Male	4720 (21.9%)	1752 (23.3%)	934 (17.7%)		905 (19.9%)		51 (17.4%)	
Overweight: Yes	8123 (37.7%)	2679 (35.7%)	1851 (35.2%)	0.550	1816 (40%)	**< 0.001**	150 (51.4%)	**< 0.001**
Educational level				**< 0.001**		**< 0.001**		**< 0.001**
Middle school	2834 (13.1%)	942 (12.5%)	785 (14.9%)		590 (13%)		51 (17.4%)	
High school	3243 (15%)	1045 (13.9%)	845 (16%)		719 (15.8%)		58 (19.8%)	
Bachelor's degree	9522 (44.1%)	3180 (42.2%)	2266 (43%)		2134 (46.9%)		112 (38.2%)	
Master's degree	5991 (27.7%)	2360 (31.4%)	1375 (26.1%)		1107 (24.3%)		72 (24.6%)	
Job categories				0.138		**< 0.001**		**< 0.001**
Higher intellectual professions	5475 (25.4%)	1962 (26.1%)	1289 (24.5%)		1104 (24.3%)		69 (23.5%)	
Intermediate professions	2903 (13.4%)	899 (11.9%)	669 (12.7%)		682 (15%)		36 (12.3%)	
Employees and workers	6425 (29.8%)	2180 (29%)	1520 (28.8%)		1448 (31.8%)		86 (29.4%)	
No professional activity	6787 (31.4%)	2486 (33%)	1793 (34%)		1316 (28.9%)		102 (34.8%)	
Comorbidities (at least one)	6444 (29.8%)	1263 (16.8%)	2032 (38.6%)	**< 0.001**	2038 (44.8%)	**< 0.001**	224 (76.5%)	**< 0.001**
Obstructive sleep apnea syndrome	2275 (10.5%)	804 (10.7%)	502 (9.5%)	0.033	475 (10.4%)	0.675	36 (12.3%)	0.384
Diagnosed	650 (3%)	169 (2.2%)	186 (3.5%)	< 0.001	177 (3.9%)	**< 0.001**	20 (6.8%)	**< 0.001**
Suspected	1788 (8.3%)	682 (9.1%)	363 (6.9%)	**< 0.001**	339 (7.5%)	**0.002**	22 (7.5%)	0.184
Diagnosed restless legs syndrome	266 (1.2%)	53 (0.7%)	91 (1.7%)	< 0.001	79 (1.7%)	**< 0.001**	11 (3.8%)	**< 0.001**
Moderate to severe depression	4049 (18.8%)	386 (5.1%)	1418 (26.9%)	**< 0.001**	1608 (35.3%)	**< 0.001**	204 (69.6%)	**< 0.001**
Sleeping drugs intake	554 (2.6%)	80 (1.1%)	277 (5.3%)	**< 0.001**	153 (3.4%)	**< 0.001**	13 (4.4%)	**< 0.001**

*Note*: Statistically significant associations are shown in bold.

^a^ *p* value for comparison between patients with insomnia without EDS and healthy participants.

^b^ *p* value for comparison between moderate sleepy insomniacs and healthy participants.

^c^ *p* value for comparison between severe sleepy insomniacs and healthy participants.

Almost half of the moderate ‘sleepy insomniacs’ and more than three‐quarters of the severe ‘sleepy insomniacs’ reported at least one comorbidity that could explain the co‐occurrence of insomnia symptoms and EDS. The most prevalent comorbidity was depression, affecting 35.3% of moderate and 69.6% of severe ‘sleepy insomniacs’. Diagnosed OSAS was more common among severe (6.8%, *p* < 0.001) and moderate (3.9%, *p* < 0.001) ‘sleepy insomniacs’ compared to healthy participants (2.2%). Suspected OSAS was less frequent among moderate ‘sleepy insomniacs’ (7.5% vs. 9.1%, *p* = 0.002). Diagnosed RLS was more prevalent in severe (3.8%, *p* < 0.001) and moderate (1.7%, *p* < 0.001) ‘sleepy insomniacs’ compared to healthy participants (0.7%). Frequent use of sleeping medications was most common among participants with insomnia symptoms without EDS (5.3%, *p* < 0.001), followed by severe (4.4%, *p* < 0.001) and moderate (3.4%, *p* < 0.001) ‘sleepy insomniacs’, compared to healthy participants (1.1%).

### 3.2 Participants Without Comorbidities (*n* = 15,146)

Half of the participants complaining of both insomnia symptoms and EDS had no comorbidities (such as OSAS, RLS, depression, or sleeping medication use). After excluding these participants, 6264 (41.4%) reported neither insomnia symptoms nor EDS, 3239 (21.4%) reported insomnia symptoms without EDS, 2512 (16.6%) were classified as moderate ‘sleepy insomniacs’, and 69 (0.5%) were classified as severe ‘sleepy insomniacs’ (Table 2). In other words, EDS (ESS ≥ 11) was reported by 44.3% of participants with insomnia symptoms (ISI ≥ 15) and 32.8% of participants without insomnia symptoms (*p* < 0.001).

**TABLE 2:** Sociodemographic characteristics, sleep complaints and sleep behaviours of participants without comorbidities according to sleep complaints.

Variables	All	Healthy participants	Insomnia symptoms without EDS	*p* ^a^	Moderate sleepy insomniacs	*p* ^b^	Severe sleepy insomniacs	*p* ^c^
Baseline, Day 1	15,146	6264	3239		2512		69	
Age (*m* ± sd)	48.7 ± 14.3	49.0 ± 15	49.5 ± 14.4	0.083	48.3 ± 13.1	**0.045**	47.8 ± 12.8	0.443
Sex				**< 0.001**		**< 0.001**		**0.049**
Female	12,243 (80.8%)	5036 (80.4%)	2747 (84.8%)		2047 (81.5%)		62 (89.9%)	
Male	2903 (19.2%)	1228 (19.6%)	492 (15.2%)		465 (18.5%)		7 (10.1%)	
Sleep complaints, Day 1	15,146	6264	3239		2512		69	
Insomnia (ISI) (*m* ± sd)	12.8 ± 5	9.4 ± 3.5	17.8 ± 2.6	**< 0.001**	17.5 ± 2.2	**< 0.001**	23.4 ± 1.7	**< 0.001**
Initial (item 1)	2769 (18.3%)	445 (7.1%)	1457 (45%)	**< 0.001**	695 (27.7%)	**< 0.001**	44 (63.8%)	**< 0.001**
Middle (item 2)	4600 (30.4%)	695 (11.1%)	1953 (60.3%)	**< 0.001**	1489 (59.3%)	**< 0.001**	59 (85.5%)	**< 0.001**
Late (item 3)	5534 (36.5%)	1116 (17.8%)	1948 (60.1%)	**< 0.001**	1648 (65.6%)	**< 0.001**	61 (88.4%)	**< 0.001**
EDS (ESS) (*m* ± sd)	9.1 ± 4.5	6.3 ± 2.7	6.2 ± 2.8	0.119	13.9 ± 2.6	**< 0.001**	18.1 ± 2.3	**< 0.001**
Passive conditions (items 1–5, 7)	8.5 ± 4	6.1 ± 2.6	6 ± 2.7	**0.031**	12.7 ± 2.1	**< 0.001**	15.6 ± 1.5	**< 0.001**
Active conditions (items 6, 8)	0.5 ± 1	0.2 ± 0.5	0.2 ± 0.5	**0.002**	1.2 ± 1.2	**< 0.001**	2.5 ± 1.4	**< 0.001**
First sleep diary, Day 1 to Day 7	2933	1083	740		569		12	
Total sleep time (*m* ± *sd*)	446 ± 48	452 ± 46	443 ± 49	**< 0.001**	440 ± 47	**< 0.001**	455 ± 66	0.895
≥ 8 h	714 (24.3%)	303 (28%)	174 (23.5%)	**< 0.001**	107 (18.8%)	**< 0.001**	4 (33.3%)	0.482
7–8 h	1365 (46.5%)	527 (48.7%)	323 (43.6%)		274 (48.2%)		4 (33.3%)	
< 7 hours	854 (29.1%)	253 (23.4%)	243 (32.8%)		188 (33%)		4 (33.3%)	
Mid‐sleep point (*m* ± *sd*)	3:24 a.m. ±71	3:25 a.m. ±70	3:30 a.m. ±75	0.149	3:16 a.m. ±66	**0.010**	3:17 a.m. ±39	0.478
Morning (earlier than 3 a.m.)	965 (32.9%)	343 (31.7%)	228 (30.8%)	0.306	221 (38.8%)	**0.006**	4 (33.3%)	0.497
Neutral (between 3 and 4 a.m.)	1288 (43.9%)	480 (44.3%)	311 (42%)		240 (42.2%)		7 (58.3%)	
Evening (later than 4 a.m.)	679 (23.2%)	260 (24%)	201 (27.2%)		108 (19%)		1 (8.3%)	
WASO (*m* ± sd)	10.3 ± 20	7.8 ± 17.4	15.0 ± 24.0	**< 0.001**	13.8 ± 22.7	**< 0.001**	16.1 ± 24.3	**0.012**
Standard deviation of WASO (*m* ± sd)	13.6 ± 30	9.9 ± 26.8	20.2 ± 34.8	**< 0.001**	19.3 ± 34	**< 0.001**	21.8 ± 37.8	**0.011**
Naps (*m* ± sd)	3.5 ± 9.3	3.4 ± 9.1	3.8 ± 10.4	0.492	3.5 ± 8	0.902	5.0 ± 6.8	0.294
Standard deviation of Naps (*m* ± sd)	1.5 ± 9.4	1.1 ± 8	1.8 ± 11.5	**< 0.007**	2.2 ± 8.5	**< 0.001**	2.8 ± 7.9	0.086
Sleep regularity index (*m* ± sd)	84.3 ± 7.4	84.9 ± 7.8	83.2 ± 6.9	**< 0.001**	84.0 ± 7.6	**0.023**	85.9 ± 3.7	0.386
Standard deviation of TST (*m* ± sd)	152 ± 66	145 ± 65	164 ± 67	**< 0.001**	156 ± 66	**0.001**	163 ± 50	0.237
Standard deviation of MSP (*m* ± sd)	106 ± 85	102 ± 84	113 ± 87	**0.007**	110 ± 102	0.081	86 ± 23	**0.044**

*Note*: Statistically significant associations are shown in bold.

^a^ *p* value for comparison between patients with insomnia without EDS and healthy participants.

^b^ *p* value for comparison between moderate sleepy insomniacs and healthy participants.

^c^ *p* value for comparison between severe sleepy insomniacs and healthy participants.

Moderate ‘sleepy insomniacs’ (48.3 years, *p* = 0.045) were significantly younger than healthy participants (49.0 years). Participants with insomnia symptoms without EDS, as well as moderate and severe ‘sleepy insomniacs’, were more likely to be female (84.8%, 81.5%, and 89.9%, respectively, vs. 80.4%, *p* < 0.001). A short TST (< 7 h) was more common among participants with insomnia symptoms without EDS (33%, *p* < 0.001) and moderate ‘sleepy insomniacs’ (33%, *p* < 0.001) compared to healthy participants (23%). The MSP occurred earlier among moderate ‘sleepy insomniacs’ (3:16 a.m., *p* = 0.010) compared to healthy participants (3:25 a.m.). Mean WASO values were higher among participants with insomnia symptoms without EDS (15.0, *p* < 0.001), and among moderate (13.8, *p* < 0.001) and severe (16.1, *p* = 0.012) ‘sleepy insomniacs’, compared to healthy participants (7.8). Reported nap duration was consistent across all groups. Sleep regularity was lower among participants with insomnia symptoms without EDS (83.2, *p* < 0.001) and moderate ‘sleepy insomniacs’ (84.0, *p* = 0.023) compared to healthy participants (84.9).

Compared to participants with insomnia symptoms without EDS, ‘sleepy insomniacs’ were younger (48.3 vs. 49.5, *p* = 0.001), more likely to be male (18.3% vs. 15.2%, *p* = 0.002), and exhibited higher rates of the late insomnia symptom (66.2% vs. 60.1%, *p* < 0.001) and lower rates of the initial insomnia symptom (28.6% vs. 45.0%, *p* < 0.001) (Table 3). They reported more EDS in both active and passive conditions. The MSP was earlier among ‘sleepy insomniacs’ (3:16 a.m., *p* = 0.001) compared to participants with insomnia symptoms without EDS (3:30 a.m.). No differences were observed in TST, WASO, nap duration, or SRI.

**TABLE 3:** Comparison of sociodemographic characteristics, sleep complaints and sleep behaviours between sleepy insomniacs and insomniacs without excessive daytime sleepiness.

Variables	Sleepy insomniacs	Insomnia symptoms without EDS	*p*
Baseline, Day 1	2581	3239	
Age (*m* ± sd)	48.3 ± 13.1	49.5 ± 14.4	**0.001**
Sex			**0.002**
Female	2109 (81.7%)	2747 (84.8%)	
Male	472 (18.3%)	492 (15.2%)	
Sleep complaints, Day 1	2581	3239	
Insomnia (ISI) (*m* ± sd)	17.7 ± 2.4	17.8 ± 2.6	**0.029**
Initial (item 1)	739 (28.6%)	1457 (45.0%)	**< 0.001**
Middle (item 2)	1548 (60.0%)	1953 (60.3%)	0.805
Late (item 3)	1709 (66.2%)	1948 (60.1%)	**< 0.001**
EDS (ESS) (*m* ± sd)	14.1 ± 2.7	6.2 ± 2.8	**< 0.001**
Passive conditions (items 1–5, 7)	12.8 ± 2.1	6.0 ± 2.7	**< 0.001**
Active conditions (items 6, 8)	1.3 ± 1.2	0.2 ± 0.5	**< 0.001**
First sleep diary, Day 1 to Day 7	581	740	
Total sleep time (*m* ± sd)	440 ± 47	443 ± 49	0.239
≥ 8 h	111 (19.1%)	174 (23.5%)	0.123
7–8 h	278 (47.8%)	323 (43.6%)	
< 7 hours	192 (33%)	243 (32.8%)	
Mid‐sleep point (*m* ± sd)	3:16 a.m. ±66	3:30 a.m. ±75	**< 0.001**
Morning (earlier than 3 a.m.)	247 (42.5%)	311 (42.0%)	**< 0.001**
Neutral (between 3 and 4 a.m.)	225 (38.7%)	228 (30.8%)	
Evening (later than 4 a.m.)	109 (18.8%)	201 (27.2%)	
WASO (*m* ± sd)	13.9 ± 22.7	15.0 ± 24	0.100
Standard deviation of WASO (*m* ± sd)	19.3 ± 34.1	20.2 ± 34.8	0.327
Naps (*m* ± sd)	3.5 ± 8.0	3.8 ± 10.4	0.617
Standard deviation of Naps (*m* ± sd)	2.3 ± 8.5	1.8 ± 11.5	0.054
Sleep regularity index (*m* ± sd)	84.0 ± 7.6	83.2 ± 6.9	0.051
Standard deviation of TST (*m* ± sd)	160 ± 65	164 ± 67	**0.025**
Standard deviation of MSP (*m* ± sd)	110 ± 101	113 ± 87	0.589

*Note: p* value for comparison between patients with insomnia without EDS and sleepy insomniacs. Statistically significant associations are shown in bold.

### 3.3 ‘Sleepy Insomniacs’ With Complete Follow‐Up Data (*n* = 434)

**FIGURE 2:**
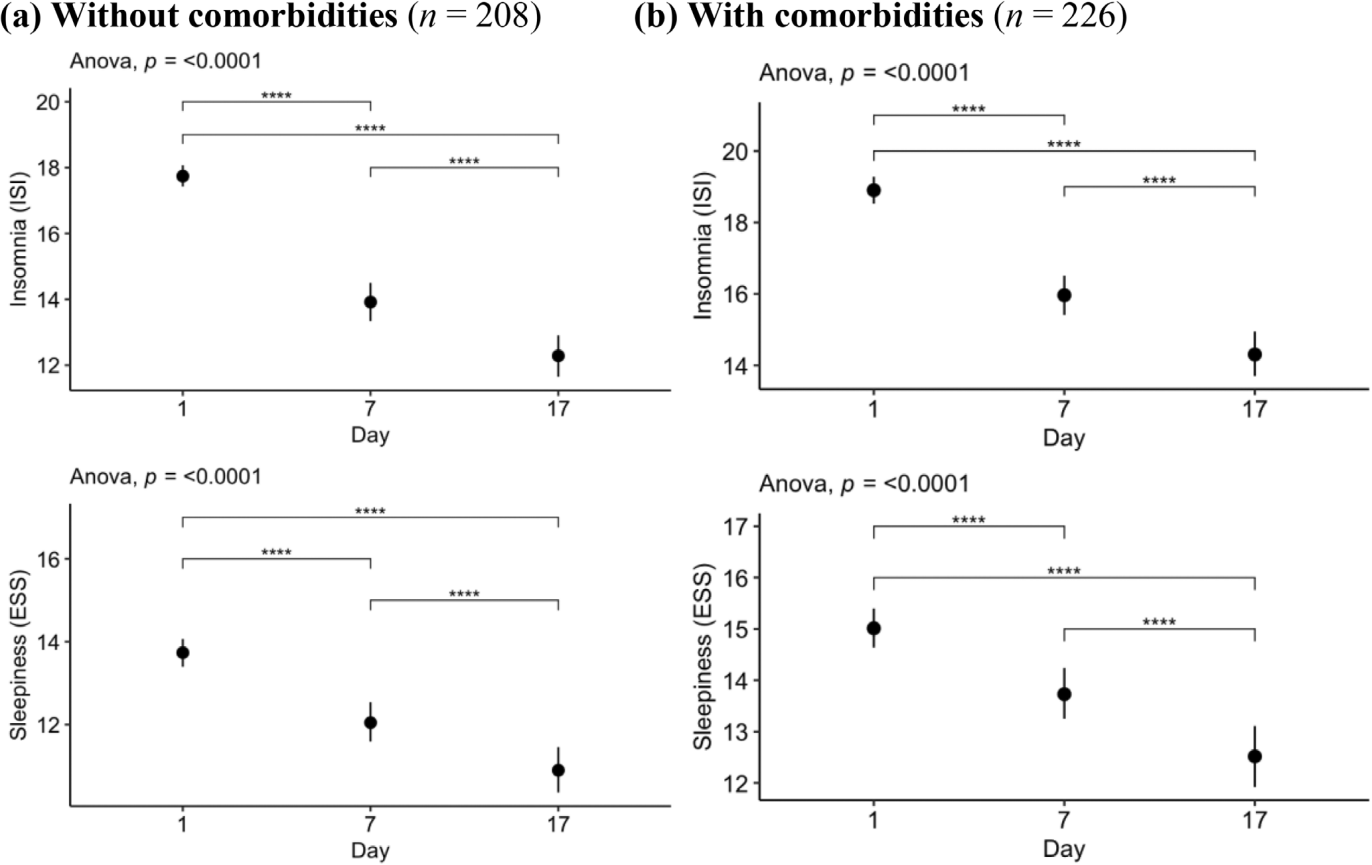
Improvement in insomnia and excessive daytime sleepiness during follow‐up among sleepy insomniacs according to comorbidities at baseline (*n* = 434). (a) Without comorbidities (*n* = 208) and (b) with comorbidities (*n* = 226). Mean and bootstrap confidence interval of insomnia (ISI) and excessive daytime sleepiness (EDS) during follow‐up at days 1, 7, and 17 among sleepy insomniacs according to comorbidities (obstructive sleep apnea, depression, restless leg syndrome, and sedative use) at baseline (*n* = 434). Panel a: sleepy insomniacs without comorbidities (*n* = 208). Panel b: Sleepy insomniacs with comorbidities (*n* = 226).

Figure 2 presents the study results. Among ‘sleepy insomniacs’ without comorbidities (*n* = 208), ISI scores decreased from 17.7 at baseline (Day 1) to 12.3 at the post‐intervention evaluation (Day 17) (*d* = 1.35, *p* < 0.001), and ESS scores decreased from 13.7 to 10.9 (*d* = 0.79, *p* < 0.001). Among ‘sleepy insomniacs’ with comorbidities (*n* = 226), ISI scores decreased from 18.9 to 14.3 (*d* = 1.11, *p* < 0.001), and ESS scores decreased from 15.0 to 12.5 (*d* = 0.62, *p* < 0.001).

## 4 Discussion

### 4.1 Key Results

In this large, nonclinical population, the phenotype of ‘sleepy insomniacs’ (ISI ≥ 15 and ESS ≥ 11) was highly prevalent, affecting 47.9% of participants with moderate‐to‐severe insomnia symptoms and 22.4% of the total population. Although a comorbidity was identified in 50% of this group (in order of frequency: depression, OSAS, sleeping drugs intake, and RLS), half of the participants complaining of both insomnia symptoms and EDS had no comorbidities. Among these participants without comorbidities, ‘sleepy insomniacs’ were more likely to be young, male, and experience middle or late insomnia symptoms compared to participants with insomnia symptoms without EDS. Interestingly, there were no significant differences in reported TST or WASO between ‘sleepy insomniacs’ and participants with insomnia symptoms without EDS, suggesting that the association between insomnia symptoms and EDS in this population is not solely driven by sleep debt. Importantly, ‘sleepy insomniacs’ showed a positive response to the digital sleep intervention, with improvements in both insomnia symptoms and EDS complaints. These findings highlight the importance of addressing EDS in the management of insomnia due to its potential comorbidities. Moreover, digital sleep interventions have demonstrated efficacy for these complaints and should be considered as a treatment option for this population.

### 4.2 A More Frequent Than Expected Phenotype

The high prevalence of ‘sleepy insomniacs’ is not surprising, as EDS is recognised as a possible daytime impairment in insomnia disorder (American Academy of Sleep Medicine 2014). However, this phenotype is unexpected when considering the most widely accepted ætiopathological hypothesis of insomnia disorder, namely the hyperarousal model (Dressle and Riemann 2023). Our findings confirm that comorbidities, as previously identified, likely explain this co‐occurrence in many cases (Sweetman et al. 2023). Nonetheless, a substantial proportion of ‘sleepy insomniacs’ had no comorbidities. Previous research on this topic has yielded inconsistent results, showing either increased (Hara et al. 2011; Johns and Hocking 1997; Kolla et al. 2020; Liu et al. 2022; Zhang et al. 2022), similar (Fasiello et al. 2024; Fortier‐Brochu et al. 2012; Lichstein et al. 1994), or decreased (Edinger et al. 2021; Roehrs et al. 2011; Sánchez‐Ortuño et al. 2011) levels of EDS in patients with insomnia compared to healthy controls (Shekleton et al. 2010). A thorough review of these studies is necessary to understand the heterogeneity of these findings. Regarding ‘sleepy insomniacs’, it has been suggested that the co‐occurrence of insomnia and EDS could be due to the variability of symptoms from day to day (i.e., no true co‐occurrence at any given moment, but rather two symptoms that alternate rapidly within an individual) (Shekleton et al. 2010). Additionally, the antagonism between arousal and EDS has been questioned (Moller et al. 2006), particularly when considering that hyperarousal is observed not only in patients with insomnia but also in those with rare hypersomnia (possibly due to chronic introversion or inhibition often seen in chronically sleepy individuals) (Pavlova et al. 2001).

### 4.3 The Role of Sleep Debt

EDS in participants with insomnia symptoms was not associated with shorter reported TST. Caution is needed when interpreting sleep behaviours reported by patients with insomnia, as they are known to have a different perception of their sleep (Valko et al. 2021). However, sleep diaries remain a widely used tool for evaluating sleep hygiene in patients with insomnia (Carney et al. 2012), as objective measurements are not yet practical in clinical settings (Nyhuis and Fernandez‐Mendoza 2023). Additionally, although WASO tends to be overestimated, TST may be closer to objective assessments (Kearns et al. 2023). Interestingly, previous studies have shown that patients with objective insomnia (i.e., those with objectively short sleep duration) were not more likely to experience increased EDS. In fact, they often reported lower EDS compared to patients with subjective insomnia (Nyhuis and Fernandez‐Mendoza 2023; Vgontzas et al. 2013). These findings challenge the hypothesis that EDS in patients with insomnia is caused by sleep debt, as was suggested in experimental studies (Bonnet and Arand 2001; Bonnet and Arand 1996).

### 4.4 The Need to Consider Insomnia Subtype

Middle and late insomnia symptoms were positively associated with EDS, whereas initial insomnia symptoms showed a negative association with EDS. These findings align with previous studies on patients with OSAS (Chung 2005) and the elderly (Yao et al. 2022). Additional details regarding the characteristics of these different insomnia subtypes can be found in Table S2. The classification of insomnia subtypes has been underexplored in recent decades due to their tendency to cluster together (Krystal 2005) and their lack of stability over time (Pillai et al. 2015). However, this could be crucial in explaining the co‐occurrence of insomnia and EDS. Although hyperarousal is typically described as a stable, 24‐h feature (Dressle and Riemann 2023), it has shown inconsistency among patients with insomnia disorder during diurnal multiple sleep latency tests (Dressle and Riemann 2023; Shekleton et al. 2010). This has led to the hypothesis that the pattern of hyperarousal across the 24‐h cycle could explain the different insomnia subtypes and the variability of EDS throughout the day (Levenson et al. 2015). Patients with diurnal hyperarousal might report reduced EDS with initial insomnia, whereas those with nocturnal hyperarousal might report increased EDS with middle or late insomnia. Other mechanisms, such as instability within the ‘flip‐flop’ switch system, might also explain the co‐occurrence of insomnia at night and EDS during the day (Levenson et al. 2015; Palagini et al. 2023). These hypotheses could be further tested by reanalysing existing data from previous studies, using objective measurements and considering insomnia subtypes. Notably, the response to our digital sleep intervention appeared to be similar across subtypes (Figure S1).

### 4.5 Treatment Response

Both insomnia symptoms and EDS complaints decreased in ‘sleepy insomniacs’ following the 17‐day digital sleep intervention, regardless of whether comorbidities were present. These results align with previous studies suggesting that digital sleep interventions are effective even in patients with comorbid sleep disorders (Araghi et al. 2013) and mental health conditions (Cunningham and Shapiro 2018). Surprisingly, EDS did not increase, as has been suggested during standard cognitive behavioural therapy for insomnia (Fasiello et al. 2024). This could be attributed to the absence of formal sleep restriction in our intervention (Haggerty et al. 2023). In fact, EDS decreased significantly, which is consistent with the results of previous trials evaluating the efficacy of various sleep interventions (Tucker et al. 2021). However, these results should be interpreted with caution due to the absence of a control group and the low follow‐up rate (9.0%), particularly among younger participants with lower levels of education, poorer sleep quality, and more severe insomnia and depressive symptoms (Sanchez Ortuño et al. 2025, 2023). Nevertheless, this high dropout rate is consistent with findings from other studies on mental health app usage, where most participants download the app but do not engage with it regularly, whether in large‐scale real‐world settings (Baumel et al. 2019; Linardon and Fuller‐Tyszkiewicz 2020) or in large randomised controlled trials (Christensen et al. 2016). Ultimately, these findings underscore the importance of considering behavioural factors in the management of EDS, both in clinical and nonclinical populations (Gandhi et al. 2021).

### 4.6 Limitations

First, distinguishing subjective EDS from fatigue, a related but distinct construct characterised by a lack of physical or mental energy rather than a tendency to fall asleep, can be particularly challenging, especially in patients with insomnia symptoms (Shen et al. 2006). However, a sensitivity analysis conducted in a large population (*n* = 14,112) using an alternative version of the KANOPÉE application demonstrated that fatigue (measured using the Fatigue Severity Scale) was not associated with insomnia subtypes in the same way as EDS. This suggests that our population was able to differentiate between these two distinct complaints (Table S3), consistent with findings in other sleep disorder patients (Suh et al. 2024). Furthermore, incorporating a broader range of sleep and mental health symptoms into network or longitudinal analyses, including closely related symptoms such as EDS, fatigue, and depressive symptoms, may provide deeper insights into the underlying mechanisms driving the co‐occurrence of insomnia and EDS. Second, although the ESS remains the most widely used and validated scale for assessing EDS, it has notable limitations that may compromise measurement accuracy. These include ambiguous items and the lack of consideration for variations in EDS throughout the day (Kendzerska et al. 2014; Miletin and Hanly 2003). In particular, the ESS focuses on sleep propensity, a subdimension of EDS, which may not be the most relevant in patients with insomnia disorder (Marques et al. 2024). Future studies adopting a multidimensional approach to EDS, incorporating other dimensions such as subjective EDS perception, but also objective assessments, are warranted. However, the weak correlations between objective and subjective measures suggest that they reflect distinct constructs (Baiardi and Mondini 2020), both of which are independently associated with functional impairments (Kosmadopoulos et al. 2017). Third, the characterisation of insomnia in this study was limited. We did not use formal diagnostic criteria for insomnia disorder, nor did we assess the duration of complaints or the extent to which they were influenced by environmental factors or consumption behaviours. Although the ISI is a widely used scale (Cerri et al. 2023) and is recommended in clinical guidelines (Riemann et al. 2017), supporting the reliability of our findings, we assessed the risk of information bias through two sensitivity analyses: one using the same sample but applying a different threshold (ISI ± 11), and another in a subsample (*n* = 2431) that benefited from a more comprehensive DSM‐5–based evaluation of insomnia disorder (including symptom frequency, duration, associated impairments and the absence of a better explanation). In both cases, we found comparable levels of EDS, as well as consistent differences in sociodemographic characteristics, sleep complaints, and sleep behaviours (Tables S4 and S5). Fourth, there was no objective measurement of comorbid sleep disorders (e.g., OSAS or periodic limb movement syndrome), nor did we assess other factors potentially associated with EDS, such as shift work, sedative treatments, cannabis or alcohol consumption, and parenting responsibilities.

## 5 Conclusion

Our findings underscore the significant prevalence of the sleepy insomniac phenotype, defined by the co‐occurrence of insomnia symptoms and EDS complaints, in a large nonclinical population. Although at least one comorbidity, primarily depression, was found in approximately half of our sample, a considerable proportion of participants without comorbidities also exhibited this unexpected phenotype. The lack of sleep debt among ‘sleepy insomniacs’ and the association with specific insomnia subtypes highlight the need for further studies to better understand the underlying mechanisms of insomnia disorder. Encouragingly, a 17‐day digital sleep intervention effectively reduced both insomnia symptoms and EDS, irrespective of comorbidities. Based on these results, we suggest that EDS should be routinely assessed in the management of participants with insomnia symptoms, and a thorough evaluation of comorbidities among ‘sleepy insomniacs’ is essential. Moreover, further studies incorporating objective measures of EDS and considering insomnia subtypes are necessary, as this specific population can benefit from digital sleep interventions.

## Author Contributions

**Julien Coelho:** conceptualization, formal analysis, methodology, visualization, writing – original draft. **Florian Pécune:** formal analysis, methodology, software, writing – original draft, writing – review and editing. **Alex Chanteclair:** writing – review and editing. **Christophe Gauld:** writing – review and editing. **Etienne de Sevin:** data curation, formal analysis, software, writing – review and editing. **Emmanuel d'Incau:** validation, writing – review and editing. **Patricia Sagaspe:** writing – review and editing. **Tafsir Ka:** writing – review and editing. **Hervé Alia:** data curation, formal analysis, software, writing – review and editing. **Charles M. Morin:** validation, writing – review and editing. **Jean‐Arthur Micoulaud‐Franchi:** conceptualization, methodology, project administration, resources, visualization, writing – original draft, writing – review and editing. **Pierre Philip:** conceptualization, data curation, funding acquisition, investigation, methodology, project administration, resources, software, supervision, validation, writing – review and editing.

## Disclosure

The authors have nothing to report.

## Ethics Statement

Informed consent was provided directly through the app and was required from all participants before any data collection could occur. Approval from the scientific committees of the University of Bordeaux was granted, and the study was in compliance with the General Data Protection Regulation, with authorisation from the French authorities (CNIL).

## Conflicts of Interest

The authors declare no conflicts of interest.

## Supporting information


**Figure S1:** Improvement in insomnia and excessive daytime sleepiness during follow‐up according to insomnia subtype at baseline (*n* = 471).
**Table S1:** Personalised behavioural recommendations from the Kanopée application.
**Table S2:** Sociodemographic characteristics and sleep behaviours of participants without comorbidities according to insomnia subtype (*n* = 15,146).
**Table S3:** Fatigue among participants without comorbidities according to insomnia subtype (*n* = 14,112).
**Table S4:** Sensitivity analysis of the comparison of sociodemographic characteristics, sleep complaints, and sleep behaviours between sleepy insomniacs and insomniacs (evaluation from the KANOPÉE application using the DSM‐5 criteria) without excessive daytime sleepiness (*n* = 1758).
**Table S5:** Sensitivity analysis of the comparison of sociodemographic characteristics, sleep complaints, and sleep behaviours between sleepy insomniacs and insomniacs (ISI ≥ 11) without excessive daytime sleepiness (*n* = 10,335).

## Data Availability

The data that support the findings of this study are available on request from the corresponding author. The data are not publicly available due to privacy or ethical restrictions.
