# Supplementary material for: Excessive Daytime Sleepiness Should Be Systematically Assessed in Individuals With Insomnia: A Population‐Based Study Employing a Virtual Agent‐Based Digital Tool
Source: J Sleep Res. 2025 Aug 28;35(2):e70178. doi: 10.1111/jsr.70178 (PMC13003272; doi:10.1111/jsr.70178)
Supplement: Supplementary file 1 — Figure S1: Improvement in insomnia and excessive daytime sleepiness during follow‐up according to insomnia subtype at baseline (n = 471). Table S1: Personalised behavioural recommendations from the Kanopée application. Table S2: Sociodemographic characteristics and sleep behaviours of participants without comorbidities according to insomnia subtype (n = 15,146). Table S3: Fatigue among participants without comorbidities according to insomnia subtype (n = 14,112). Table S4: Sensitivity analysis of the comparison of sociodemographic characteristics, sleep complaints, and sleep behaviours between sleepy insomniacs and insomniacs (evaluation from the KANOPÉE application using the DSM‐5 criteria) without excessive daytime sleepiness (n = 1758). Table S5: Sensitivity analysis of the comparison of sociodemographic characteristics, sleep complaints, and sleep behaviours between sleepy insomniacs and insomniacs (ISI ≥ 11) without excessive daytime sleepiness (n = 10,335). [file JSR-35-e70178-s001.docx]

**Supplementary Table 1.** Personalized behavioral recommendations from the Kanopée application

| **List of the recommendations** | | |
| --- | --- | --- |
| N°1 | Keep a fixed wake up schedule every day of the week, even on weekends, to train your biological clock. | |
| N°2 | Keep a reasonable amount of time in bed to reinforce the bed/sleep association: If I am awake for more than 15 minutes at night, I get out of bed and only come back to bed when I am sleepy | |
| N°3 | In the morning: exposing yourself to sunshine or strong light (light therapy, screens) ensures that your biological clock works properly. | |
| N°4 | Physical activity at home will complement the light to keep your sleep/wake rhythm stable. To practice ideally 1 hour in the morning and to refrain activity 3 to 4 hours before bedtime. | |
| N°5 | Do not shift your bedtime and wake up times and do not hang out in bed in the morning even if the night has been bad. | |
| N°6 | Moderate the consumption of stimulants (coffee, coke, energy drinks): no more than 4 cups or glasses per day and do not drink any more after 2 p.m. | |
| N°7 | Go to bed only when you are sleepy. Avoid reading and snacking in bed. | |
| N°8 | In the evening, do not skip dinner but avoid too fatty dishes and favor starchy foods (which prevents nighttime cravings). | |
| N°9 | Disconnect 1h to 2h before going to bed (turn off smartphone, tablet, computer) and leave the screens off until the next morning. | |
| N°10 | Create a bedroom conducive to sleep: darkness, silence and a temperature between 18°C ​​and 20°C. | |
| N°11 | Be careful, you may be sleep deprived, try to increase your time spent in bed to 7 hours per night | |
| **Recommendation personalization algorithm** | | |
| **IF**  ISI Q1 ≥ 4 | | N°2 (stimulus control)  N°3 (light)  N°7 (stimulus control) |
| **IF**  ISI Q2 ≥ 4  **OR**  ≥ 1 WASO ≥ 15’ | | N°2 (stimulus control)  N°4 (physical activity)  N°5 (regularity)  N°6 (stimulants consumption)  N°10 (bed environment) |
| **IF**  Efficacy > 85% **AND**  TST > 7 hours | | N°1 (regularity)  N°3 (light)  N°4 (physical activity)  N°5 (regularity) |
| **IF**  Efficacy > 85%  **AND**  ISI > 21  **AND**  TST < 6 hours | | N°11 (increased sleep duration) |
| **ELSE** | | N°1 (regularity)  N°2 (stimulus control)  N°3 (light)  N°4 (physical activity)  N°6 (stimulants consumption)  N°8 (diner)  N°9 (screen)  N°10 (bed environment) |
| ***ISI****: Insomnia Severity Index.* ***WASO****: Wake After Sleep Onset.* ***TST****: Total Sleep Time*. | | |

**Supplementary Table 2**. Sociodemographic characteristics and sleep behaviors of participants without comorbidities according to insomnia subtype (*n* = 15,146)

| Variables | **All** | **Healthy participants** | **Initial insomnia** | ***p*** | **Middle insomnia** | ***p*** | **Late**  **insomnia** | ***p*** | **Combined insomnia** | ***p*** |
| --- | --- | --- | --- | --- | --- | --- | --- | --- | --- | --- |
| Baseline, Day 1 | 15,146 | 9,326 | 720 |  | 856 |  | 1,424 |  | 2,820 |  |
| **Age** (*m* ± *sd*) | 48.7 ± 14.3 | 48.5 ± 14.6 | 47.2 ± 15.8 | **0.040** | 49.2 ± 12.3 | 0.106 | 46.3 ± 14 | **<0.001** | 50.6 ± 13.4 | **<0.001** |
| **Sex** |  |  |  | **<0.001** |  | **<0.001** |  | 0.245 |  | **<0.001** |
| - Female | 12243 (80.8%) | 7387 (79.2%) | 627 (87.1%) |  | 737 (86.1%) |  | 1147 (80.5%) |  | 2345 (83.2%) |  |
| - Male | 2903 (19.2%) | 1939 (20.8%) | 93 (12.9%) |  | 119 (13.9%) |  | 277 (19.5%) |  | 475 (16.8%) |  |
| **Insomnia** (ISI) (*m* ± *sd*) | 12.8 ± 5 | 9.7 ± 3.4 | 17.5 ± 2.3 | **<0.001** | 17.2 ± 2.2 | **<0.001** | 17.2 ± 2.1 | **<0.001** | 18.3 ± 2.7 | **<0.001** |
| - Initial (item 1) | 2769 (18.3%) | 573 (6.1%) | 704 (97.8%) | **<0.001** | 66 (7.7%) | 0.071 | 150 (10.5%) | **<0.001** | 1276 (45.2%) | **<0.001** |
| - Middle (item 2) | 4600 (30.4%) | 1099 (11.8%) | 136 (18.9%) | **<0.001** | 829 (96.8%) | **<0.001** | 338 (23.7%) | **<0.001** | 2198 (77.9%) | **<0.001** |
| - Late (item 3) | 5534 (36.5%) | 1877 (20.1%) | 94 (13.1%) | **<0.001** | 143 (16.7%) | **0.016** | 1402 (98.5%) | **<0.001** | 2018 (71.6%) | **<0.001** |
| **EDS** (ESS) (*m* ± *sd*) | 9.1 ± 4.5 | 8.7 ± 4.3 | 8.3 ± 4.7 | **0.025** | 10 ± 4.5 | **<0.001** | 10.5 ± 4.7 | **<0.001** | 9.5 ± 4.8 | **<0.001** |
| - Passive conditions (items 1-5, 7) | 8.5 ± 4 | 8.2 ± 3.8 | 7.8 ± 4.2 | **0.005** | 9.4 ± 4 | **<0.001** | 9.7 ± 4 | **<0.001** | 8.9 ± 4.2 | **<0.001** |
| - Active conditions (items 6, 8) | 0.5 ± 1 | 0.5 ± 0.9 | 0.5 ± 0.9 | 0.196 | 0.6 ± 1 | **<0.001** | 0.8 ± 1.1 | **<0.001** | 0.7 ± 1 | **<0.001** |
| First sleep diary, Day 1 to Day 7 | 2,933 | 1,612 | 144 |  | 222 |  | 360 |  | 595 |  |
| **Total sleep time** (*m* ± *sd*) | 446 ± 48 | 450 ± 48 | 439 ± 52 | **0.013** | 454 ± 43 | 0.245 | 440 ± 50 | **<0.001** | 439 ± 48 | **<0.001** |
| - ≥ 8 hours | 714 (24.3%) | 429 (26.6%) | 29 (20.1%) | 0.090 | 59 (26.6%) | 0.324 | 78 (21.7%) | **0.007** | 119 (20%) | **<0.001** |
| - 7-8 hours | 1365 (46.5%) | 764 (47.4%) | 67 (46.5%) |  | 115 (51.8%) |  | 160 (44.4%) |  | 259 (43.5%) |  |
| - < 7 hours | 854 (29.1%) | 419 (26%) | 48 (33.3%) |  | 48 (21.6%) |  | 122 (33.9%) |  | 217 (36.5%) |  |
| **Mid-sleep point** (*m* ± *sd*) | 3:24 a.m. ± 71 | 3:24 a.m. ± 72 | 4:05 a.m. ± 70 | **<0.001** | 3:16 a.m. ± 77 | 0.152 | 3:18 a.m. ± 60 | 0.080 | 3:21 a.m. ± 72 | 0.364 |
| - Morning (earlier than 3 a.m.) | 965 (32.9%) | 512 (31.8%) | 22 (15.3%) | **<0.001** | 81 (36.5%) | 0.217 | 132 (36.7%) | 0.172 | 218 (36.6%) | 0.098 |
| - Neutral (between 3 and 4 a.m.) | 1288 (43.9%) | 730 (45.3%) | 53 (36.8%) |  | 100 (45%) |  | 156 (43.3%) |  | 249 (41.8%) |  |
| - Evening (later than 4 a.m.) | 679 (23.2%) | 369 (22.9%) | 69 (47.9%) |  | 41 (18.5%) |  | 72 (20%) |  | 128 (21.5%) |  |
| **WASO*** (IIM) (*m* ± *sd*) | 10.3 ± 20 | 7.4 ± 16.6 | 11.3 ± 19.3 | **<0.001** | 20.6 ± 28 | **<0.001** | 9.9 ± 19 | **<0.001** | 15.7 ± 24.2 | **<0.001** |
| **WASO*** (ISD) (*m* ± *sd*) | 13.6 ± 30 | 9.6 ± 26.1 | 16.4 ± 32.3 | **<0.001** | 26.9 ± 38.5 | **<0.001** | 13.9 ± 28.7 | **<0.001** | 21.5 ± 35.8 | **<0.001** |
| **Naps**** (IIM) (*m* ± *sd*) | 3.5 ± 9.3 | 3.4 ± 9.5 | 5 ± 10.3 | 0.059 | 3.2 ± 6.8 | 0.612 | 3.6 ± 9.3 | 0.795 | 3.6 ± 9.6 | 0.745 |
| **Naps**** (ISD) (*m* ± *sd*) | 1.5 ± 9.4 | 1.3 ± 8.8 | 2.3 ± 11.8 | **0.020** | 2.0 ± 7.6 | **0.009** | 2.1 ± 9.5 | **0.001** | 1.8 ± 10.9 | **0.021** |
| **Sleep regularity index** (*m* ± *sd*) | 84.3 ± 7.4 | 84.8 ± 7.6 | 82.3 ± 7.4 | **<0.001** | 84.1 ± 7.3 | 0.173 | 83.5 ± 7.3 | **0.002** | 83.8 ± 7 | **0.002** |

**Supplementary Table 3**. Fatigue among participants without comorbidities according to insomnia subtype (*n* = 14,112)

| Variables | **All** | **Healthy participants** | **Initial insomnia** | ***p*** | **Middle**  **insomnia** | ***p*** | **Late**  **Insomnia** | ***p*** | **Combined insomnia** | ***p*** |
| --- | --- | --- | --- | --- | --- | --- | --- | --- | --- | --- |
| Baseline, Day 1 | 14,112 | 4,609 | 1,183 |  | 1,263 |  | 2,751 |  | 4,306 |  |
| **Age** (*m* ± *sd*) | 47.7 ± 15.2 | 49.0 ± 15.9 | 44.7 ± 17.2 | **<0.001** | 47.6 ± 13.2 | **0.001** | 45.5 ± 15.1 | **<0.001** | 48.5 ± 14.1 | 0.069 |
| **Sex** |  |  |  | **<0.001** |  | **<0.001** |  | **<0.001** |  | **<0.001** |
| Female | 9,009 (63.8%) | 2,605 (56.5%) | 825 (69.7%) |  | 919 (72.8%) |  | 1,763 (64.1%) |  | 2,897 (67.3%) |  |
| Male | 5,103 (36.2%) | 2,004 (43.5%) | 358 (30.3%) |  | 344 (27.2%) |  | 988 (35.9%) |  | 1,409 (32.7%) |  |
| **Fatigue** (*m* ± *sd*) | 4.6 ± 1.4 | 3.7 ± 1.4 | 5.0 ± 1.1 | **<0.001** | 4.9 ± 1.1 | **<0.001** | 5.0 ± 1.1 | **<0.001** | 5.0 ± 1.2 | **<0.001** |

**Supplementary Table 4**. Sensitivity analysis of the comparison of sociodemographic characteristics, sleep complaints, and sleep behaviors between sleepy insomniacs and insomniacs (evaluation from the KANOPÉE application using the DSM-5 criteria) without excessive daytime sleepiness (*n* = 1,758).

| Variables | **Sleepy insomniacs** | **Insomnia symptoms**  **without EDS** | ***p*** |
| --- | --- | --- | --- |
| Baseline, Day 1 | 738 | 1,020 |  |
| **Age** (*m* ± *sd*) | 50.8 ± 13.1 | 51.5 ± 14.5 | 0.335 |
| **Sex** |  |  | **0.004** |
| - Female | 546 (74.0%) | 727 (71.3%) |  |
| - Male | 192 (26.0%) | 293 (28.7%) |  |
| Sleep complaints, Day 1 | 738 | 1,020 |  |
| **Insomnia** (ISI) (*m* ± *sd*) | 17.3 ± 4.0 | 16.8 ± 4.3 | **0.004** |
| - Initial (item 1) | 291 (39.4%) | 519 (50.9%) | **<0.001** |
| - Middle (item 2) | 435 (58.9%) | 542 (53.1%) | **0.016** |
| - Late (item 3) | 426 (57.7%) | 498 (48.8%) | **<0.001** |
| **EDS** (ESS) (*m* ± *sd*) | 14.0 ± 2.8 | 6.1 ± 2.8 | **<0.001** |
| - Passive conditions (items 1-5, 7) | 12.8 ± 2.2 | 5.9 ± 2.7 | **<0.001** |
| - Active conditions (items 6, 8) | 1.2 ± 1.2 | 0.2 ± 0.4 | **<0.001** |
| First sleep diary, Day 1 to Day 7 | 204 | 273 |  |
| **Total sleep time** (*m* ± *sd*) | 439 ± 55 | 439 ± 49 | 0.994 |
| - ≥ 8 hours | 41 (20.1%) | 59 (21.6%) | 0.878 |
| - 7-8 hours | 88 (43.1%) | 119 (43.6%) |  |
| - < 7 hours | 75 (36.8%) | 95 (34.8%) |  |
| **Mid-sleep point** (*m* ± *sd*) | 3:25 a.m. ± 59 | 3:35 a.m. ± 76 | 0.133 |
| - Morning (earlier than 3 a.m.) | 76 (37.3%) | 76 (27.8%) | **0.05** |
| - Neutral (between 3 and 4 a.m.) | 82 (40.2%) | 114 (41.8%) |  |
| - Evening (later than 4 a.m.) | 46 (22.5%) | 83 (30.4%) |  |
| **Sleep regularity index** (*m* ± *sd*) | 84.5 ± 7.4 | 83.8 ± 8.2 | 0.375 |
| **Standard deviation of TST** (*m* ± *sd*) | 153 ± 66 | 159 ± 71 | 0.381 |
| **Standard deviation of MSP** (*m* ± *sd*) | 100 ± 53 | 117 ± 117 | **0.030** |
| *p-value* for comparison between participants with insomnia without EDS and sleepy insomniacs | | | |

**Supplementary Table 5**. Sensitivity analysis of the comparison of sociodemographic characteristics, sleep complaints, and sleep behaviors between sleepy insomniacs and insomniacs (ISI ≥ 11) without excessive daytime sleepiness (*n* = 10,335).

| Variables | **Sleepy insomniacs** | **Insomnia symptoms**  **without EDS** | ***p*** |
| --- | --- | --- | --- |
| Baseline, Day 1 | 4,313 | 6,022 |  |
| **Age** (*m* ± *sd*) | 48.2 ± 13.2 | 49.5 ± 14.5 | **<0.001** |
| **Sex** |  |  | **<0.001** |
| - Female | 3460 (80.2%) | 4987 (82.8%) |  |
| - Male | 853 (19.8%) | 1035 (17.2%) |  |
| Sleep complaints, Day 1 | 2,581 | 3,239 |  |
| **Insomnia** (ISI) (*m* ± *sd*) | 15.6 ± 3.2 | 15.4 ± 3.3 | **0.029** |
| - Initial (item 1) | 844 (19.6%) | 1834 (30.5%) | **<0.001** |
| - Middle (item 2) | 1910 (44.3%) | 2557 (42.5%) | 0.065 |
| - Late (item 3) | 2326 (53.9%) | 2783 (46.2%) | **<0.001** |
| **EDS** (ESS) (*m* ± *sd*) | 14.0 ± 2.6 | 6.3 ± 2.7 | **<0.001** |
| - Passive conditions (items 1-5, 7) | 12.7 ± 2.1 | 6.1 ± 2.7 | **<0.001** |
| - Active conditions (items 6, 8) | 1.2 ± 1.2 | 0.2 ± 0.5 | **<0.001** |
| First sleep diary, Day 1 to Day 7 | 581 | 740 |  |
| **Total sleep time** (*m* ± *sd*) | 440 ± 47 | 443 ± 49 | **0.028** |
| - ≥ 8 hours | 174 (19.8%) | 317 (24.7%) | **0.026** |
| - 7-8 hours | 418 (47.6%) | 580 (45.2%) |  |
| - < 7 hours | 287 (32.7%) | 385 (30.0%) |  |
| **Mid-sleep point** (*m* ± *sd*) | 3:16 a.m. ± 66 | 3:30 a.m. ± 75 | **<0.001** |
| - Morning (earlier than 3 a.m.) | 326 (37.1%) | 393 (30.7%) | **<0.001** |
| - Neutral (between 3 and 4 a.m.) | 385 (43.8%) | 544 (42.4%) |  |
| - Evening (later than 4 a.m.) | 167 (19.0%) | 345 (26.9%) |  |
| **Sleep regularity index** (*m* ± *sd*) | 84.3 ± 7.3 | 83.5 ± 7.5 | **0.023** |
| **Standard deviation of TST** (*m* ± *sd*) | 154 ± 64 | 160 ± 67 | **0.028** |
| **Standard deviation of MSP** (*m* ± *sd*) | 106 ± 88 | 112 ± 95 | 0.191 |
| *p-value* for comparison between participants with insomnia without EDS and sleepy insomniacs | | | |

**Supplementary Figure 1**. Improvement in insomnia and excessive daytime sleepiness during follow-up according to insomnia subtype at baseline (*n* = 471)

**(a) Initial insomnia** (*n* = 53) **(b) Middle insomnia** (*n* = 77) **(c) Late insomnia** (*n* = 129) **(d) Combined insomnia** (*n* = 212)


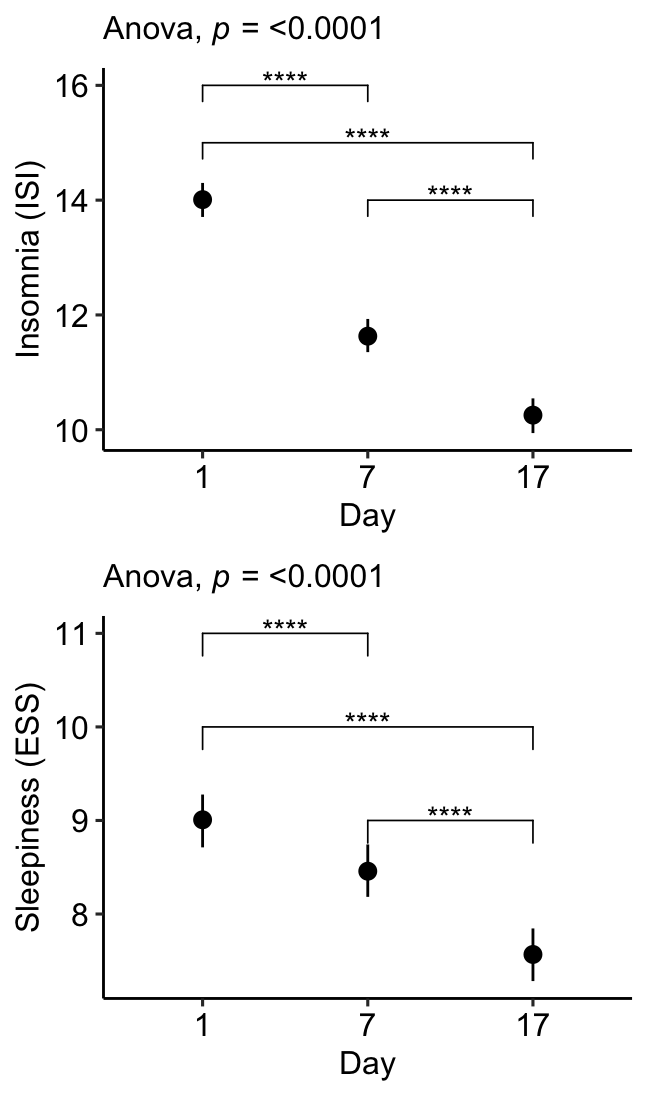

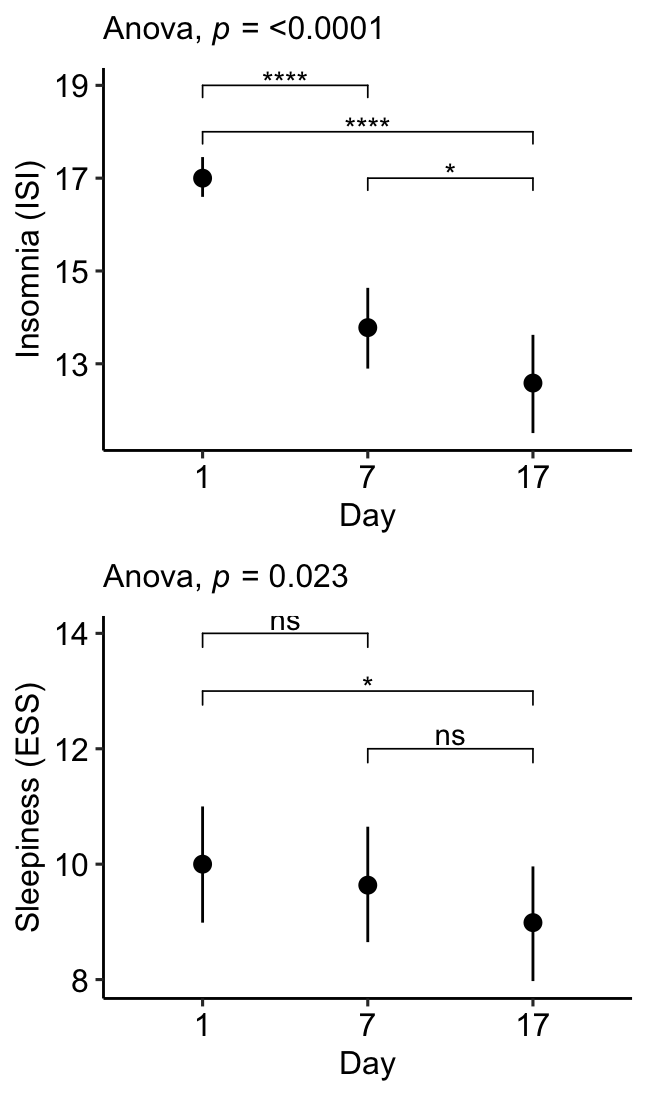

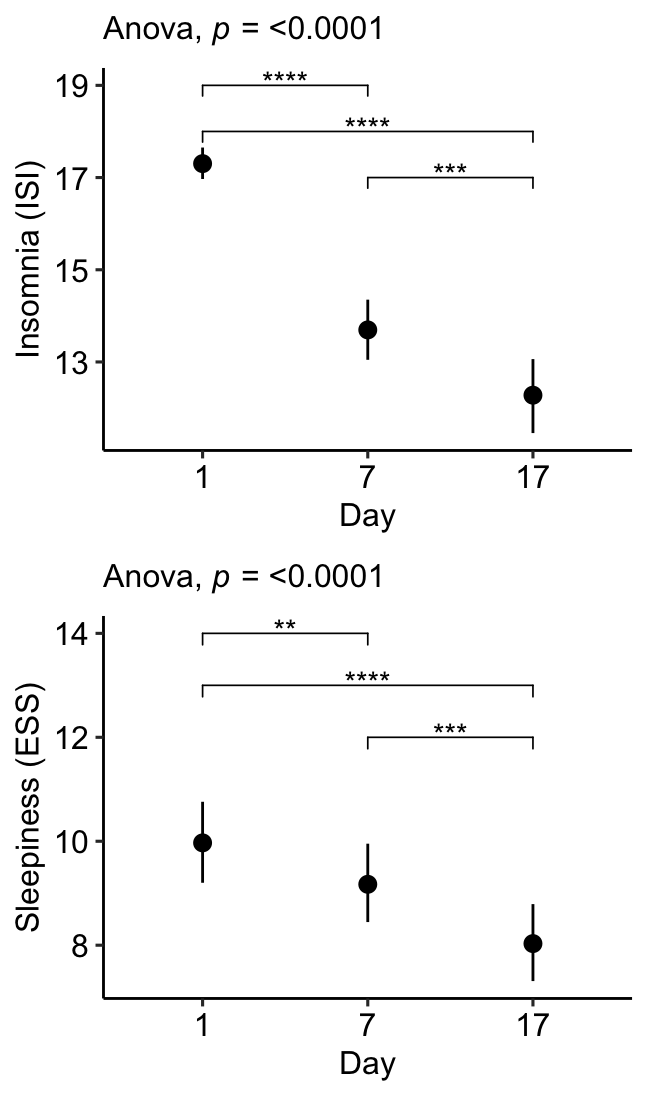

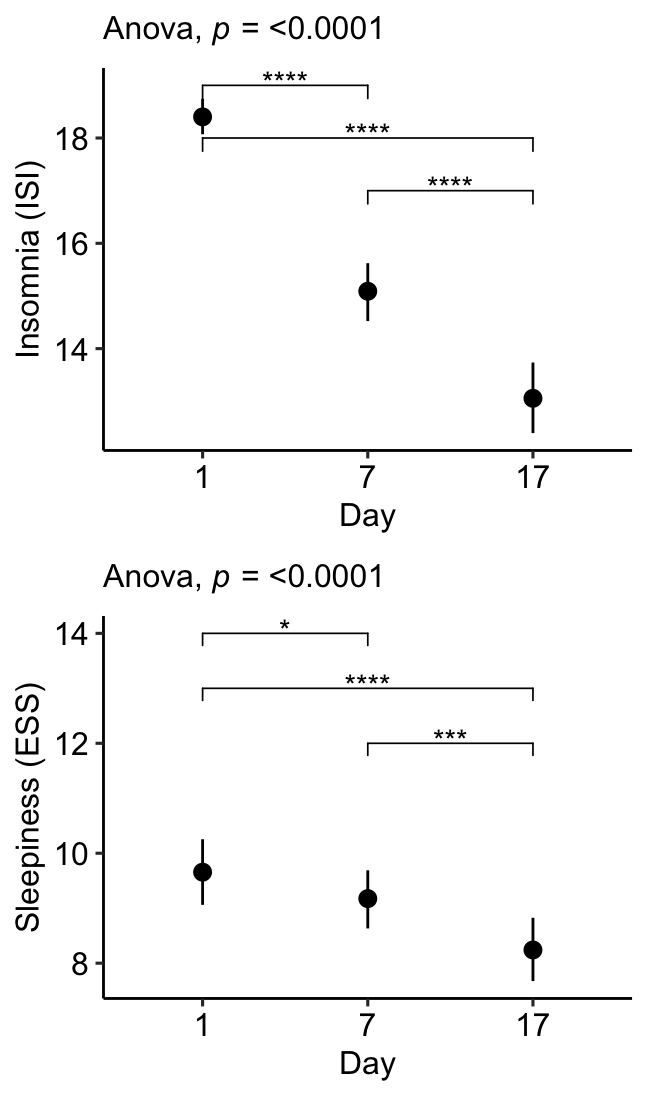


***Supplementary Figure 1.*** *Mean and bootstrap confidence interval of insomnia (ISI) and excessive daytime sleepiness (EDS) during follow-up at days 1, 7, and 17 according to insomnia subtype at baseline (n = 471).* ***Panel a:*** *initial insomnia (n = 53).* ***Panel b****: middle insomnia (n = 77).* ***Panel c****: late insomnia (n = 129).* ***Panel d****: combined insomnia (n = 212).*
